# Hermetic Bags Effectively Manage Emerging and Common Pests of Stored Cowpeas in Niger

**DOI:** 10.3390/insects16020196

**Published:** 2025-02-11

**Authors:** Habibou Yahaya Dan Bawa, Ibrahim Boukary Baoua, Mahamane Moctar Rabé, Dieudonne Baributsa

**Affiliations:** 1 Faculté d’Agronomie et des Sciences de l’Environnement, Université Dan Dicko Dankoulodo de Maradi, Maradi BP 465, Niger; habibyahaya34@gmail.com (H.Y.D.B.); baoua.ibrahim@gmail.com (I.B.B.); 2 Department de Sociologie et Economie Rurale, Université de Tahoua, Tahoua BP 255, Niger; mocnad1@gmail.com; 3 Department of Entomology, Purdue University, West Lafayette, IN 47907, USA

**Keywords:** post-harvest loss, cowpea bruchids, smallholder farmers, airtight storage, Sahel

## Abstract

The cowpea is a vital crop for low-resource farmers in the Sahel, but post-harvest losses due to insect pests remain a major challenge. *Callosobruchus maculatus* (Fabricius, 1775), is the primary pest responsible for most of the damage to stored cowpeas. Recently, *Trogoderma granarium* (Everts, 1898) was found infesting cowpeas in large warehouses in Niger. This study evaluated hermetic storage bags to manage both common and emerging insect pests. Treatments included (i) the Purdue Improved Crop Storage (PICS) hermetic bag; (ii) a woven polypropylene (PP) bag with a polyethylene (PE) liner and Phostoxin; and (iii) a woven PP bag without Phostoxin (control). Naturally infested cowpea grains were obtained from the Office des Produits Vivriers du Niger (OPVN) warehouse in Maradi, Niger. Infestation levels were assessed using 12 samples of 500 g each, randomly collected from each treatment at the start and end of the trial. Major pests identified were *C. maculatus*, *T. granarium*, and *Tribolium* sp., with initial populations of 0.83, 0.44, and 0.83 adults per 500 g of cowpea, respectively. After six months of storage, pest densities in the control increased significantly: 232-fold for *C. maculatus*, 7.4-fold for *T. granarium*, and 2.7-fold for *Tribolium* sp.; resulting in a 38.5% weight loss. In contrast, both the Phostoxin and the PICS hermetic bags effectively suppressed pest populations, preventing weight loss. This study confirms the efficacy of hermetic storage, such as the PICS bag, in protecting cowpeas from both common and emerging pests.

## Simple Summary

The cowpea is a crucial crop for low-resource farmers in the Sahel, but insect pests like *Callosobruchus maculatus* cause significant post-harvest losses. Recently, *Trogoderma granarium* was detected in cowpea storage in Niger. This study evaluated the effectiveness of hermetic storage bags (Purdue Improved Crop Storage- PICS) compared to woven polypropylene (PP) bags with or without insecticides. The insecticide treatment had a polyethylene liner inside the PP bag. Infested cowpeas from warehouses in Maradi, Niger, were monitored for six months. Pest populations increased drastically in the control, resulting in a 38.5% weight loss. However, PICS bags and insecticides successfully suppressed pests, preventing any weight loss. The findings confirm that hermetic storage is highly effective in protecting cowpeas from common and emerging pests.

## 1. Introduction

In West Africa, particularly Niger, the cowpea is the most important legume and a primary protein source for rural populations [1,2,3]. Effective post-harvest management is vital for food security, as family farms depend on their production to meet basic nutritional needs [4,5]. Urban communities typically access cereal and legume grains through storage systems located near production areas in Niger or via imports from neighboring countries [6]. Storage losses are caused mainly by *Callosobruchus maculatus* (Fabricius, 1775), the primary pest responsible for most of the damage to stored cowpea [7,8]. Another species, *Trogoderma granarium* (Everts, 1898), historically associated with groundnut stocks in Niger’s Maradi and Zinder regions [9], has recently been reported on millet and cowpea stored in warehouses, signaling its expanding presence and threat to stored crops.

*Trogoderma granarium* is a globally recognized and serious pest of cereals and stored products worldwide, particularly in tropical and subtropical regions [10]. Native to the Indian subcontinent, it has spread widely through trade to Africa, Asia, Europe, and South America [11,12]. Its economic impact stems from the significant damage caused by its larvae, which consume grains voraciously, often leaving only the husks behind. The larvae also hide in cracks and small spaces, where they can enter diapause for months or even years [12,13,14]. This ability not only enhances their survival but also facilitates their spread when conditions for population growth become favorable. Unlike many pests, *T. granarium* cannot fly, relying entirely on human activity for dispersal. The primary mode of its spread is the international transport of infested goods, underscoring the need for strict trade rules and storage practices to mitigate its impact.

Controlling *T. granarium* is challenging due to its biological and behavioral characteristics. Methyl bromide fumigation is commonly used for eradication when infestations are detected [15]. However, methyl bromide has been banned in most countries due to its ozone-depleting properties [16]. In Niger, traders and food security agencies, including the Office des Produits Vivriers du Niger (OPVN) and the United Nations (UN) World Food Program (WFP), frequently report *T. granarium* infestations in large warehouses storing cereals and legumes. These organizations raised concerns about the inefficacy of widely used pesticides. Resistance to synthetic pesticides has become a major issue, driven by repeated chemical applications and the pest’s ability to enter diapause, enabling it to survive adverse conditions [14,17,18,19].

Alternative management strategies for *T. granarium* have been explored as supplements or substitutes for traditional chemical treatments. These include temperature control in storage facilities, high-pressure and temperature treatments under laboratory settings, entomopathogenic fungi, entomotoxic proteins, and modified atmosphere storage using carbon dioxide under high pressure [10,16,20,21,22]. While some of these methods have shown promise, their practical application in field conditions remains limited. In contrast, hermetic storage bags have been tested and proven effective in controlling various stored product pests, including cowpea bruchids [23,24]. This study evaluated the potential of hermetic storage to manage *T. granarium*, an emerging threat to cowpea stocks in food security agencies’ warehouses.

## 2. Materials and Methods

### 2.1. Experiment Setup

The study was conducted in the laboratory of the Biodiversity Center at the Dan Dicko Dankoulodo University of Maradi (UDDM), located in the south-central region of Niger. The experiment ran from 25 August 2023 to 25 February 2024. Treatments consisted of (i) hermetic Purdue Improved Crop Storage (PICS) bag; (ii) ordinary woven polypropylene (PP) bag fitted with a 25-micron-thick polyethylene (PE) liner and treated with the fumigant aluminum phosphide (Phostoxin); and (iii) ordinary woven PP bag as a control. For each insecticide treatment, a Phostoxin tablet wrapped in tissue paper was inserted into the bag before sealing. Cowpea grains, naturally infested and sourced from Office des Produits Vivriers du Niger (OPVN), were used in this experiment. The cowpea had been stored in the OPVN’s warehouse for three years. The grains were thoroughly mixed to ensure uniform infestation and then divided into three treatments, with 40 kg per bag and four replicates per treatment, totaling 12 bags. The 50-kg bags (PICS, PP, and PE) used for storing cowpeas were purchased from local input vendors. After filling, the bags were kept at ambient temperature and relative humidity for the duration of the experiment.

### 2.2. Data Collection and Analysis

Cowpea infestation was evaluated using three 500 g samples randomly collected from each replicate at the start and end of the trial. Each 500 g sample was sifted to remove impurities and separate larvae and adult insects of all pest species. Only larvae of *T. granarium* were assessed in the 500 g samples, as they are external feeders and easily identifiable. From each of the three 500 g samples, three 100-grain subsamples were taken, resulting in a total of 900 grains per replicate (*n* = 36 per treatment). These 100-grain subsamples were used to assess grain damage and weight loss. Insect-damaged kernels and weight loss were determined as follows:

$$\%\;infested\;grains=\frac{Number\;of\;infested\;grains}{Total\;number\;of\;grains}\times 100$$

$$\%\;weight\;loss=\frac{\left(Final\;weight-Initial\;weight\right)}{Initial\;weight}\times 100$$

After assessing the grain damage and weight loss, the 100-grain subsamples were further examined for the presence of *C. maculatus* larvae, as these are internal feeders. The subsamples were soaked in water for 24 h, then the grains were split open to count the larvae. Oxygen (O_2_) and carbon dioxide (CO_2_) levels in the PICS bags were measured every three days for the first two months using a Mocon PAC Check Model 325 Headspace Analyzer (Mocon, Minneapolis, MN, USA). Unfortunately, the equipment malfunctioned after two months, preventing data collection for the remaining four months of the experiment.

Statistical analyses were conducted using the Statistical Package for Social Science (SPSS) software, version 22.0. Analysis of variance (ANOVA), followed by the Student-Newman-Keuls test (SNK) test, was used to compare the means of insect counts, weight loss, damaged grains, and grains with holes. When the data did not follow a normal distribution, the Kruskal-Wallis test was used to compare group means.

## 3. Results

Throughout the experiment, the room’s relative humidity ranged from 21.16% to 67.73%, while the temperature varied between 26.18 °C and 37.89 °C. The Kruskal-Wallis tests showed significant differences in oxygen (H = 20.10, *p* < 0.0001) and carbon dioxide (H = 20.46, *p* < 0.0001) concentrations within the PICS bags. The decrease in oxygen levels was minimal (3.6%), while the increase in CO_2_ levels was significant (Table 1).

The mean impurity weights for each treatment were as follows: 25.68 ± 6.93 for the initial; 36.99 ± 13.24 for Phostoxin; 34.12 ± 13.64 for PICS bags; and 56.49 ± 17.19 for the control. The Kruskal-Wallis test indicated a significant difference in impurity weights across treatments and control (H = 32.68, *p* < 0.0001). At the start of the experiment, impurities accounted for 5.14% of the total weight of 500 g (Table 2). After six months of storage, this proportion increased to 7.4% and 6.8% in the Phostoxin and PICS bag treatments, respectively. In contrast, impurities in the control treatment rose significantly to 11.3%. Three insect pest species were identified on cowpea: *T. granarium*, *C. maculatus*, and *Tribolium* sp. The initial adult populations were 0.83, 0.44, and 0.83 insects per 500 g for *T. granarium*, *C. maculatus*, and *Tribolium* sp., respectively. After six months in the control treatment, adult insect infestations increased by 7.4-fold *T. granarium*, 232-fold for *C. maculatus*, and 2.7-fold for *Tribolium* sp. Compared to the initial, no changes were observed in adult *T. granarium*, *C. maculatus*, and *Tribolium* sp. populations in Phostoxin and PICS bag treatments.

Data collected from 100 grains revealed an average of 3.06 *C. maculatus* larvae per sample at the start of the experiment (Table 3). After six months, this number remained unchanged in the Phostoxin and PICS bag treatments. However, in the control treatment, the number of larvae increased by 19-fold. The initial weight of 100 seeds was 14.34 g, which remained stable in the Phostoxin and the PICS bag treatments after six months of storage. In contrast, the control treatment experienced a 38.5% weight loss compared to the baseline.

**Figure 1:**
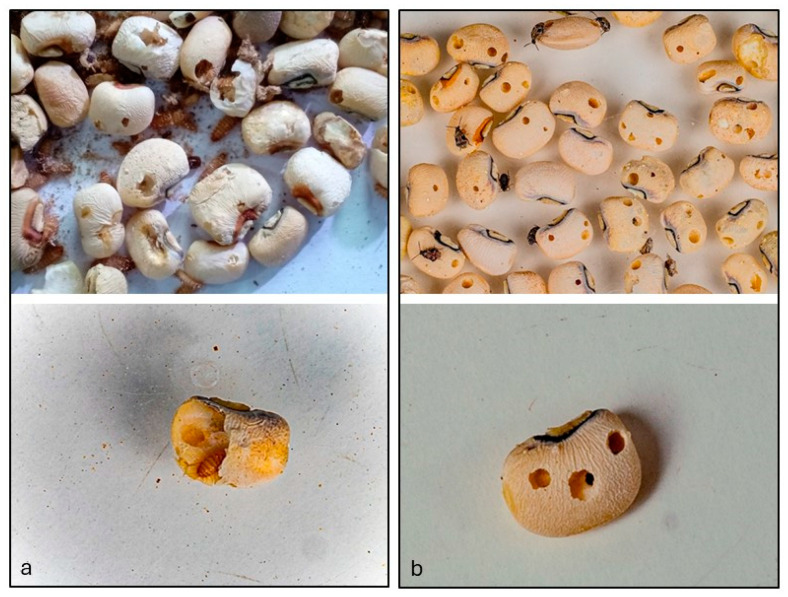
Images depicting the damage caused by (**a**) *Trogoderma granarium* and (**b**) *Callosobrocus maculatus* on single and multiple cowpea grains stored for six months in woven polypropylene bags in Maradi, Niger. Damage by *C. maculatus* is characterized by emergence holes, whereas *T. granarium* causes damage through external feeding.

Damage by *C. maculatus* was characterized by emergence holes, whereas *T. granarium* caused damage through external feeding (Figure 1). Compared to the initial value, the percentage of seeds damaged by *T. granarium* remained unchanged in the Phostoxin and the PICS bag treatments. However, this rate increased 1.5-fold in the control bag after six months of storage. After six months, grain damage by *C. maculatus* in the Phostoxin treatment remained consistent with the initial values. In contrast, it increased 1.58-fold in the PICS bag treatment and 18.11-fold in the control compared to the beginning of the experiment.

## 4. Discussion

*Callosobrocus maculatus* is the most common pest affecting stored cowpeas [4,8,25]. In West Africa, *C. maculatus* was previously considered the sole insect pest species on stored cowpeas [5,26], with no reports of other pests. A survey of smallholder farmers identified *T. granarium* exclusively in stored sorghum in Niger [27]. This study is one of the first to report the presence of *T. granarium* in stored cowpeas. Typically found in storage facilities, warehouses, and mills [12,13,28], *T. granarium* poses a significant risk to commodities stored in large warehouses prone to invasive pests [29].

*Trogoderma granarium* is a polyphagous quarantine pest mainly spread through human activities, especially international trade [13,16,30]. Once established, it is difficult to control due to its ability to infest and reproduce in various commodities, its facultative larval diapause, and insecticide resistance [31,32,33]. The presence of *T. granarium* in Niger’s large warehouses, which handle food aid products from multiple countries and locally purchased grain, creates ideal conditions for pest infestations. These stocks are stored for several years before sales or distributions. This likely explains its attacks on cowpeas. Shifts in a species’ food preferences are common and often driven by complex interactions between post-harvest and other ecosystems [34]. This study also found *Tribolium* sp., another polyphagous pest of minor importance as it mainly feeds on damaged seeds [35,36].

The data revealed three pest species with varying infestation levels and impacts. Initial infestation of *T. granarium* was similar to that of other pests but became less prolific after six months of storage compared to *C. maculatus* in the untreated control. The initial high *T. granarium* larvae population may be due to its ability to survive repeated chemical treatments. *Callosobrocus maculatus* grew exponentially in the control, reflecting its resilience as a common pest of stored cowpeas. Meanwhile, *Tribolium* sp. populations showed a minimal increase. Both hermetic PICS bags and insecticide treatments effectively halted the development of all three pests. Previous studies have shown that hermetic storage bags and phosphine fumigation control pests such as cowpea bruchids, *Rhizopertha dominica* (Fabricius, 1792), and *Tribolium castaneum* (Herbst, 1797) [6,23,24,37,38,39,40,41].

The combined effects of the three pest species caused significant damage, leading to higher impurity levels and considerable weight loss in cowpeas stored in woven PP bags (control). Similar weight losses of 16.7% to 52% after 4.5 to 8 months of storage have been reported in Niger [23,24,42]. These findings highlight cowpea’s high vulnerability to pests during storage [25,43,44], emphasizing the need for preservation measures. Damages by *C. maculatus* and *T. granarium* increased over time in control, while hermetic PICS bags and phosphine fumigation (Phostoxin) effectively minimized losses, reducing adult and larval densities. These findings align with prior research demonstrating the effectiveness of chemical controls and hermetic bags in managing cowpea bruchids [23,24,45,46,47,48]. Hermetic storage is thus a reliable and sustainable solution for reducing pest-related losses in stored cowpeas while limiting chemical exposure.

Although phosphine (Phostoxin) is effective, reports of pesticide resistance are increasing. In Burkina Faso, *C. maculatus* strains showed greater resistance to cypermethrin and pirimiphos-methyl than those in Senegal and Niger [48]. Similarly, *T. granarium*, especially in its diapausing larval stage, is resistant to phosphine and other pesticides [14,18,32,49]. The repeated use of chemicals has led to resistance, health risks, and food poisoning [45], highlighting the need for sustainable pest control methods. Hermetic storage offers a promising alternative by creating hypoxic and hypercarbia conditions that kill pests, achieving mortality rates similar to phosphine. While insect presence in cowpeas stored in hermetic bags is rare, it warrants further investigation. Further research is needed to assess the impact of hermetic storage on different pest life stages, including larvae, adults, and eggs.

## 5. Conclusions

This study on cowpeas stored in large warehouses identified three key insect pests: *C. maculatus*, *T. granarium*, and *Tribolium* sp. While *C. maculatus* remains the dominant pest, the presence of *T. granarium* marks a shift in pest dynamics, likely driven by inter-ecosystem interactions and anthropogenic factors such as international trade. These findings highlight the susceptibility of cowpeas to both existing and emerging pests, leading to significant losses in weight and quality. Hermetic storage technologies, such as PICS bags, effectively manage this emerging pest and provide a viable alternative to chemical treatments, addressing both rising insecticide resistance and food safety concerns. Further research is needed to understand the behavior of *T. granarium* across life stages under hermetic storage conditions and optimize storage methods for managing diapausing stages. This study offers valuable insights for developing integrated pest management strategies to safeguard stored cowpeas and enhance food security.

## Figures and Tables

**Table 1:** Oxygen (O_2_) and carbon dioxide (CO_2_) concentration (±SEM) in naturally infested cowpeas stored in the Purdue Improved Crop Storage (PICS) bag for two months.

Period	*n*	Oxygen Concentration (%) *	Carbon Dioxide Concentration (%)
Initial	10	20.72 ± 0.33 a	0.13 ± 0.26 b
After 1 month	9	18.58 ± 1.41 b	4.93 ± 2.12 a
After 2 months	10	17.12 ± 1.33 b	7.53 ± 3.47 a

*All means ± standard error of means (SEM). Means within the same column followed by the same letter are not significantly different at *p* > 0.05.

**Table 2:** Average number (±SEM) of live insect species in 500 g of cowpea grains naturally infested and stored for six months.

Period	Treatment	*n*	*T. granarium* Larvae	*T. granarium* Adults	*C. maculatus* Adults	*Tribolium* sp. Adults
Initial		36	19.28 ± 8.18 b*	0.83 ± 1.05 b	0.44 ± 0.74 b	0.83 ± 1.16 b
After 6 months	Phostoxin	12	0.50 ± 0.91 c	0.00 ± 0.00 b	0.25 ± 0.62 b	0.08 ± 0.29 b
	PICS bag	12	0.67 ± 1.50 c	0.17 ± 0.58 b	2.75 ± 1.55 b	0.00 ± 0.00 b
	Control	12	52.33 ± 20.19 a	6.17 ± 3.38 a	102.0 ± 30.78 a	2.25 ± 2.90 a
	ANOVA		F = 70.94; *p* < 0.001	F = 43.65; *p* = 0.003	F = 221.60; *p* < 0.001	F = 6.33; *p* < 0.001

* All data are means ± standard error of means (SEM). Means within the same column followed by the same letter are not significantly different at *p* > 0.05.

**Table 3:** Infestation, weight, and damage by *C. maculatus* and *T. granarium* per 100 grains of naturally infested cowpea stored for six months.

Period	Treatment	*n*	Number of *C. maculatus* Larvae in 100 Grains	Weight of 100 Grains	Number of Grains Damaged by *T. granarium*	Number of Grains with *C. maculatus* Holes
Initial		108	3.06 ± 1.8 b*	14.34 ± 0.95 a	7.44 ± 3.05 b	4.77 ± 3.05 c
After 6 months	Phostoxin	36	2.42 ± 2.28 b	14.28 ± 0.93 a	7.14 ± 2.95 b	4.81 ± 2.17 c
	PICS bag	36	2.00 ± 1.35 b	14.38 ± 0.70 a	6.58 ± 3.06 b	7.53 ± 3.36 b
	Control	36	58.33 ± 19.49 a	8.82 ± 1.42 b	10.94 ± 3.69 a	86.42 ± 10.39 a
	ANOVA		F = 474.49; *p* < 0.001	F = 298.58; *p* < 0.001	F = 2614.04; *p* < 0.001	F = 12.47; *p* < 0.001

* All data are means ± standard error of means (SEM). Means within the same column followed by the same letter are not significantly different (*p* > 0.05).

## Data Availability

Raw data are not publicly available but may be obtained upon request.
